# Characterization of the patterns of drug-resistance mutations in newly diagnosed HIV-1 infected patients naïve to the antiretroviral drugs

**DOI:** 10.1186/1471-2334-9-111

**Published:** 2009-07-16

**Authors:** Claudia Alteri, Valentina Svicher, Caterina Gori, Roberta D'Arrigo, Massimo Ciccozzi, Francesca Ceccherini-Silberstein, Marina Selleri, Stefano Aviani Bardacci, Massimo Giuliani, Paola Elia, Paola Scognamiglio, Roberta Balzano, Nicoletta Orchi, Enrico Girardi, Carlo Federico Perno, SENDIH Study Group

**Affiliations:** 1 Department of Experimental Medicine and Biochemical Sciences, University of "Tor Vergata" Rome, Italy; 2 National Institute for Infectious Diseases "L. Spallanzani", Rome, Italy; 3 Department of Infectious Parasite and Immuno-Mediate Diseases, Istituto Superiore di Sanità, Rome, Italy; 4 AIDS Center, Belcolle Hospital, Viterbo, Italy; 5 Division of Dermatological Infectious Diseases, STI/HIV Unit, San Gallicano Institute, Rome, Italy

## Abstract

**Background:**

The transmission of HIV-1 drug-resistant strains in drug naive patients may seriously compromise the efficacy of a first-line antiretroviral treatment. To better define this problem, a study in a cohort of newly diagnosed HIV-1 infected individuals has been conducted. This study is aimed to assess the prevalence and the patterns of the mutations recently associated with transmitted drug resistance in the reverse transcriptase (RT) and in protease (PR) of HIV-1.

**Methods:**

Prevalence of transmitted drug resistant strains is determined in 255 newly diagnosed HIV-1 infected patients enrolled in different counselling and testing (CT) centres in Central Italy; the Avidity Index (AI) on the first available serum sample is also used to estimate time since infection. Logistic regression models are used to determine factors associated with infection by drug resistant HIV-1 strains.

**Results:**

The prevalence of HIV-1 strains with at least one major drug resistance mutation is 5.9% (15/255); moreover, 3.9% (10/255) of patients is infected with HIV nucleoside reverse transcriptase inhibitor (NRTI)-resistant viruses, 3.5% (9/255) with HIV non-NRTI-resistant viruses and 0.4% (1/255) with HIV protease inhibitor (PI)-resistant viruses. Most importantly, almost half (60.0%) of patients carries HIV-1 resistant strains with more than one major drug resistance mutation. In addition, patients who had acquired HIV through homosexual intercourses are more likely to harbour a virus with at least one primary resistance mutation (OR 7.7; 95% CI: 1.7–35.0, P = 0.008).

**Conclusion:**

The prevalence of drug resistant HIV-1 strains among newly diagnosed individuals in Central Italy is consistent with the data from other European countries. Nevertheless, the presence of drug-resistance HIV-1 mutations in complex patterns highlights an additional potential risk for public health and strongly supports the extension of wide genotyping to newly diagnosed HIV-1 infected patients.

## Background

The development of resistance to the currently available antiretroviral drugs against HIV-1 infection is one of the major limitations to the maintenance of a successful treatment. Its frequent detection among HIV-infected treatment failing patients [1-3] can in turn increase the risk of new infections driven by drug-resistant viral strains [4]. This can carry important clinical implications. Indeed, once transmitted, a drug-resistant virus can persist for months to years without reversion to wild-type [5]. In addition, the presence of drug-resistant HIV-1 strains in drug naïve patients is associated with an increased probability of virological failure to the first-line antiretroviral therapy [6-8]. For these reasons, the new guidelines recommend to perform the genotypic resistance testing in all drug naive patients, before beginning a first line antiretroviral regimen [9,10].

To date, there is a growing literature about the rate of transmission of HIV-1 drug-resistant virus. In the United States and in Europe, where there is a wide access to highly active antiretroviral therapy (HAART), the prevalence of HIV-1 drug-resistant strains ranges between 3.3% and 14.0% in recently infected patients and between 6.1% and 12.5% in chronically infected ones [11-16].

The estimation of the rates within the patterns of transmitted drug resistance mutations is crucial for surveillance programmes and for providing feedback on the efficacy of HIV-1 prevention strategies.

To better define the size of the phenomenon, we led a study in a cohort of newly diagnosed HIV-1 infected patients in Central Italy aimed at defining i) the prevalence of the classical as well as of the novel mutations recently associated with resistance to RT and PR inhibitors (and their correlation with viral-immunological parameters), ii) the clusters of drug resistance mutations and iii) the circulation of HIV-1 subtypes and putative recombinant forms (CRFs).

## Methods

### Study population

The study included 263 HIV-1 infected individuals enrolled between January 2004 and March 2007 in the SENDIH (Studio Epidemiologico Nuove Diagnosi Infezione HIV-1) programme, a multicenter study aimed to collect behavioural, virological and molecular data on persons with newly diagnosed HIV infection. Characteristics and methods of the study have been previously described elsewhere [17]. Individuals with a first HIV-1 positive test performed in 10 public Counselling and Testing centres (CTC) in Lazio Region, Italy, were invited to participate in the study. At the diagnosis, clinical and immunologic data, and blood sample have been collected from all participants to investigate the molecular characterization of the virus and to identify recently acquired infections.

Informed consent was obtained from participants and the ethics committee of the National Institute for Infectious Diseases L. Spallanzani, Rome approved the study. All of the information gathered during the study was analyzed in a completely anonymous way.

### The IgG avidity assay

To estimate time since infection, we calculated the Avidity Index (AI) on the first available serum sample, using an automated anti-HIV enzyme immunoassay (EIA), as previously described [18-20]. The method is based on the rationale that antibodies produced in the early phase of an infection show a low avidity for the antigen, and the antibody avidity increases progressively with the time after exposure to an immunogen. Thus, a low avidity is likely to indicate a recent infection. In particular, an AI < 0.80 has been reported to fairly define an infection acquired in the 6 months prior the diagnosis (recent infection) while AI ≥ 0.80 is generally used in literature to define long-standing infection [18-20]. Misclassifications of recent infections as long-standing infections (and vice versa) could not be excluded for patients with an AI index between 0.80 and 0.90 [18].

### HIV sequencing

HIV genotype analysis was performed on plasma samples by means of a commercially available kit (ViroSeq HIV-1 genotyping system; Abbott Laboratories) [21]. The polymerase chain reaction was performed in all the 263 samples and was successful for 255 samples (yeld 97%), that were then sequenced (yeld of 97%). Briefly, RNA was extracted, retrotranscribed by murine leukemia virus reverse transcriptase (RT), and amplified with Amplitaq-Gold polymerase enzyme by using two different sequence-specific primers for 40 cycles. *Pol*-amplified products (containing the entire protease and the first 335 amino acids of the reverse transcriptase open reading frame, 1302 nt) were full-length sequenced in sense and antisense orientations by an automated sequencer (ABI 3100) by using seven different overlapping sequence-specific primers [21]. Sequences having a mixture of wild-type and mutant residues at single positions were considered to have the mutant(s) at that position.

### Phylogenetic analysis

All 255 HIV-1, sequences (1302 nt) were aligned and compared with reference sequences for the Major HIV-1 subtypes, available at: http://www.hiv.lanl.gov/content/sequence/NEWALIGN/align.html using CLUSTAL X [22]. The sequences were then manually edited with the Bioedit program [23], and gaps were removed from the final alignment. All sequences were analyzed using the REGA HIV-1 subtyping tool. [24]

Separate trees were then generated using F84 Model of substitution with both NJ and Maximum Likelihood (ML) tree building methods [25], for both non-B pure subtypes and putative recombinant forms.

Phylogenetic trees were performed with different evolutionary model according to the Hierarchical Likelihood Ratio Test (HLRT) implemented in the Model Test V3.0 software [26]. The statistical robustness within each phylogenetic tree was confirmed with a bootstrap analysis using 1000 replicates for the Neighbor-Joining (NJ) tree. All calculations were performed with PAUP*4.0 software [25].

Simplot software version 3.2 [27] was used to generate similarity plots and bootscan plots, for genetic diversity and intersubtype recombination analysis.

### Determination of drug resistance mutations

To estimate the prevalence of resistant strains, we used the list of drug resistance mutations associated with transmitted drug resistance, that is reported by Shafer et al., 2007 [28]. This list is used in all the epidemiological and surveillance studies addressing the transmission of HIV drug-resistance in drug naïve patients: in the RT, M41L, K65R, D67N/G, D67del, T69D, T69ins, K70R, L74V, V75A/M/T/S, F77L, L100I, K101E, K103N/S, V106A/M, Y115F, F116Y, Q151M, Y181C/I, M184V/I, Y188C/H/L, G190A/E/S/Q, L210W, T215C/D/E/F/I/S/Y/V, K219E/Q/R, P225H, M230L, P236L; in the PR, L24I, D30N, V32I, M46I, I47A/V, G48V, I50L/V, F53L, I54A/L/M/S/T/V, G73A/C/S/T, V82A/F/M/T/S, I84A/C/V, N88D/S, L90M [28,29].

We also determined the prevalence of the RT polymorphism V60I that has been associated with the persistence of tymidine analogues mutations 1 (TAMs1) in drug naïve patients [30].

Other polymorphisms at positions already associated with drug resistance were also investigated.

### Statistical analysis

#### (i) Quantitative measurements and mutation prevalence

For quantitative measurements, data sets with non-normal distributions were compared non-parametrically using Mann-Whitney U test. Categorical data were analyzed by using Fisher exact test. A p value less than 0.05 was used to determine statistical significance. Logistic regression analysis was used to examine the association between epidemiological, clinical and virological factors.

#### (ii) Mutation covariation

We calculated the binomial correlation coefficient (phi) for all the possible pairwise combinations between all mutations related with drug resistance. The covariation analysis was performed in the 213 HIV-1 B subtype infected patients.

All calculations were performed using a script implemented in the R software, version 2.7.1 http://www.r-project.org.

Statistically significant pairwise correlations were those with a P value < 0.05. For each pair, two positions each with a mixture of two or more mutations were excluded from the covariation analysis, since it is impossible to discriminate whether these mutations fall in the same viral genome. We used the Benjamini-Hochberg method to identify pairwise combinations that were significant in the presence of multiple-hypothesis testing; a false discovery rate of 0.05 was used to determine statistical significance. In order to analyze the covariation structure of mutations in more detail, we performed mutational clusters, defined as clusters of three or more mutated positions in which each position was significantly correlated with each other, identified by a computational technique that evaluated all possible clusters that can be formed from the significant correlated pairwise combinations of mutated positions [31].

## Results

### Study population

Table 1 summarizes the main characteristics of the 255 out of 263 newly diagnosed patients, whose samples were successfully amplified and sequenced. Epidemiological information was available for 249 out of 255 individuals.

**Table 1:** Distribution of HIV-1 drug resistance mutations by selected demographic, clinical and virological characteristics of 255 newly diagnosed patients

Characteristics	All patients n = 255	Patients with at least one drug resistance mutation (NRTI, NNRTI, PI)^d^ n = 15	Patients with wild type virus n = 240	*P-value*^*e*^	OR (95% CI)^f^
**Italian^a ^N (%)**	188 (75.5)	14 (93.3)	174 (74.3)	0.13	4.8 (0.6–37.5)
**Male^a ^N (%)**	193 (77.5)	14 (93.3)	179 (76.5)	0.13	4.8 (0.6–37.5)
**Median age (years) (IQR)^a^**	37 (31–43)	38 (34–46)	37 (30–43)	0.35	
**Risk exposure N (%)**					
**Heterosexual**	108 (47.6)	2 (13.3)	106 (50.0)	0.01	0.15 (0.03–0.70)
**Homosexual**	110 (48.6)	13 (86.7)	97 (45.8)	**0.008**	**7.7 (1.7–35.0)**
**Intravenous drug use**	9 (4.0)	0 (0.0)	9 (4.2)	1.00	0.0 (0.0–6.1)
**Unknown, no**.	28	0	28		
**Viral Load, Median (IQR), ** **(log copies/ml)**	4.9 (4.3–5.4)	4.5 (4.3–5.2)	4.9 (4.3–5.4)	0.40	
**CD4+ count, Median (IQR), cell/mm3**	372 (171–517)	336 (132–450)	385 (194–527)	0.50	
**Co-infection N (%)**					
**HBV**	33 (13.3)	0 (0.0)	33 (13.7)	0.23	0.0 (0.0–1.6)
**HCV**	26 (10.4)	0 (0.0)	26 (10.8)	0.38	0.0 (0.0–2.2)
**HIV-1 subtypes**					
**Subtype B**	213 (83.5)	15 (100)	198 (82.5)	0.99	inf (2.2-inf)
**Other Subtypes**	42 (16.5)	0 (0.0)	42 (17.5)	0.99	0.0 (0.0–1.2)
**Infectious status**					
**Long-standing Infection,** ** > 6 months^b^**	197 (77.2)	13 (86.7)	184 (76.7)	0.38	2.0 (0.4–9.0)
**Recent Infection, ≤ 6 months^c^**	58 (22.7)	2 (13.3)	56 (23.3)	0.38	0.50 (0.11–2.3)

^a ^Among 255 patients, epidemiological information was available for 249 patients. The 6 patients without epidemiological information are included in the group of 240 patients carried wild type virus. Thus, the percentages were calculated on 249 patients in the second column and on 234 patients on the forth column.

^b ^Long-standing infection was estimated by a ≥ 0.80 avidity index.

^c ^Recent infection was estimated by a < 0.80 avidity index.

^d ^Mutations that have been associated with transmitted drug resistance (Shafer et al., 2007) are taking into account.

^e ^For quantitative measurements, data sets with non-normal distributions were compared non-parametrically using Mann-Whitney U test. Categorical data were analyzed by using a chi-square test accomplished by a logistic regression analysis.

^f ^CI, confidence interval, odds ratio (OR) are calculated with a logistic regression analysis

One hundred ninety three (193, 77.5%) individuals were males and more than 75% of patients were Italian (n = 188). Among 61 foreigners, 25 patients originated from South America (41.0%), 17 from Sub-Saharan Africa (27.9%), 14 from Eastern Europe (23.0%) and 5 from North and Central America (8.2%).

Based on results of Avidity Index, 58 (22.7%) patients were classified as recent infected, while the remaining 197 (77.2%) were classified as long-standing infected patients. At the time of median diagnosis, CD4 cell count was higher in individuals with a recent infection compared to those with a long-standing infection (480 [interquartile range, IQR: 366–655] cell/mm3 vs 330 [IQR: 146–484] cell/mm3, P < 0.001, data not shown) while median viremia was not significantly different in the two groups of patients (4.8 [IQR: 4.0–5.4] log10 HIV-1 RNA copies/ml vs 4.9 [IQR: 4.4–5.4] log10 HIV-1 RNA copies/ml, P = 0.09). A major proportion of Italian patients was found in the group of recently HIV-1 infected patients than in the long-standing infected one (49 [87.5%] vs 139 [72.0%], P = 0.02, data not shown).

Several factors that might affect the risk of becoming infected with drug-resistant virus were explored (Table 1). Patients who had acquired HIV through homosexual intercourses were more likely to harbour a virus with at least one primary resistance mutation (OR 7.7; 95% CI: 1.7–35.0, P = 0.008). In particular, homosexuals had a significant increased risk of harbouring HIV with resistance mutations to NRTI (OR 9.5, 95% CI: 1.2–76.6, P = 0.03), while risk of resistance mutations to NNRTI and PI was not significantly associated with HIV risk factor (P = NS). This may reflect the higher and earlier access to treatment of this class than other groups of patients.

### Circulation of HIV-1 subtypes in newly diagnosed patients in Italy

**Figure 1:**
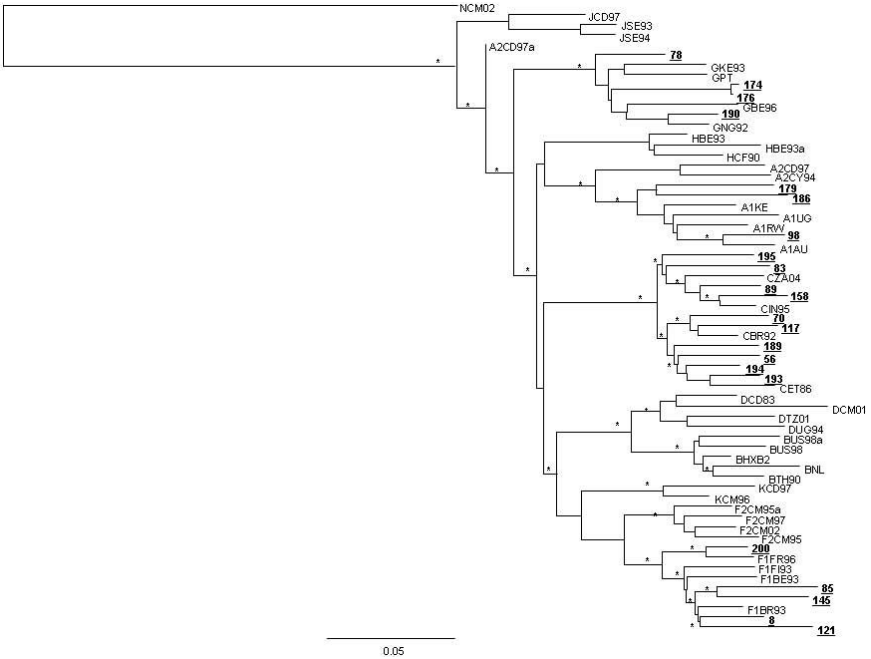
**Phylogenetic relationships based on *pol *gene (1302 nt) of the HIV-1 *pol *gene between the HIV-1 non-B pure subtypes circulating in Italy (shown in bold and underlined) and representative strains of HIV-1 M group (subtypes A, C, D, F1, F2, G, H, J, K) from the Los Alamos HIV Sequence Database**. The scale bar indicates 5% nucleotide sequence divergence.* indicates the *P value *< 0.001 (zero length branch test) and the bootstrap values more than 70%.

**Figure 2:**
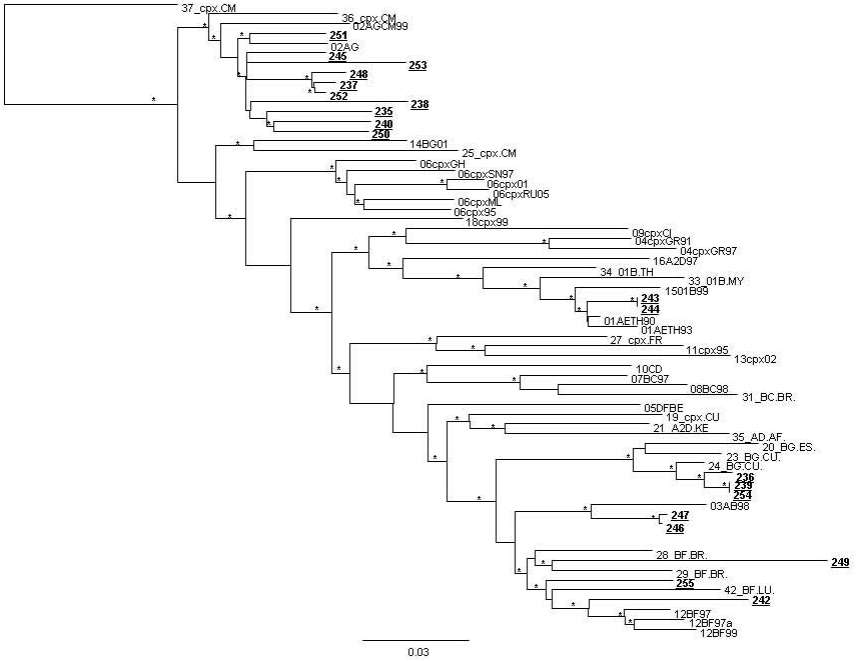
**Phylogenetic relationships on *pol *gene (1302 nt) of the HIV-1 *pol *gene between the HIV-1 putative recombinant forms circulating in Italy (shown in bold and underlined) and the reference sequences of the CRFs of the HIV-1 M group from the Los Alamos HIV Sequence Database**. Bootstrap values <90% are not shown. The scale bar indicates 3% nucleotide sequence divergence.* indicates the *P value *< 0.001 (zero length branch test) and the bootstrap values more than 70%.

The figures 1 and 2 represent the 22 non-B pure subtypes and the 20 putative recombinant forms circulating in Italy.

Phylogenetic analysis showed that B subtype was the most predominant in the overall population (213 [83.5%]), followed by C subtype and CRF02_AG (10/255 [3.92%] each one). Regarding the country of origin, the majority of patients infected by B subtype was Italian (170 [79.8%]). Among the 42 individuals infected with a non-B subtype, 18 (42.9%) were Italian, 7 (16.7%) were from a European country, and 17 (40.5%) were from outside Europe. The prevalence of C and A subtypes was higher in non-Italian compared to Italian patients (7 [11.4%] vs 3 [1.6%], P = 0.003, and 3 vs 0 [0.0%], P = 0.01). Thus, nearly half of patients infected with a non-B subtypes were Italian.

Interestingly, we also observed the presence of CRF03_AB in one recently and in one long-standing infected patients, coming from Italy and Eastern Europe, respectively. This is the first study that reports the circulation of CRF03_AB in Italy (Figure 2).

### Prevalence of major mutations associated with drug resistance

Among the 255 newly diagnosed, 15 (5.9%) carried HIV-1 strains with at least 1 major mutation associated with transmitted drug resistance (Table 2) [28,29]; all of them were infected with subtype B.

**Table 2:** Prevalence of resistant HIV-1 strains

Mutation^a^	Prevalence			Infectious status		
		
	Total^b^		in combination^c^	Long-standing Infection	Recent Infection	
					
	All patients	Subtype B	N (%)	> 6 months (N = 197)	≤ 6 months (N = 58)	*P-value*^*d*^
	N (%/255)	N (%/213)				
**Major Drug resistance mutations**						
Any	15 (5.9)	15 (7.0)	9 (60.0)	13 (6.6)	2 (3.4)	0.09
**NRTI**						
Any	10 (3.9)	10 (4.7)	8 (80.0)	8 (4.1)	2 (3.4)	1
Any TAM	7 (2.6)	7 (3.3)	7 (100)	6 (3.0)	1 (1.7)	1
Any TAM1	7 (2.6)	7 (3.3)	7 (100)	6 (3.0)	1 (1.7)	1
Any TAM2	1 (0.4)	1 (0.5)	1 (100)	0 (0.0)	1 (1.7)	0.2
M41L	4 (1.6)	4 (1.9)	4 (100)	3 (1.5)	1 (1.7)	1
D67N	1 (0.4)	1 (0.5)	1 (100)	0 (0.0)	1 (1.7)	0.2
T69D	1 (0.4)	1 (0.5)	0 (0.0)	0 (0.0)	1 (1.7)	0.2
M184V	1 (0.4)	1 (0.5)	1 (100)	1 (0.5)	0 (0.0)	1
L210W	6 (2.3)	6 (2.8)	6 (100)	5 (2.5)	1 (1.7)	1
T215Y	2 (0.8)	2 (0.9)	2 (100)	1 (0.5)	1 (1.7)	0.4
T215D/S	8 (3.1)	8 (3.8)	6 (75.0)	8 (4.1)	0 (0.0)	0.2
**NNRTI**						
Any	9 (3.5)	9 (4.2)	5 (55.5)	8 (4.1)	1 (1.7)	0.7
L100I	2 (0.8)	2 (0.9)	2 (100)	2 (1.0)	0 (0.0)	1
K101E	2 (0.8)	2 (0.9)	0 (0.0)	2 (1.0)	0 (0.0)	1
K103N	7 (2.7)	7 (3.3)	5 (71.4)	6 (3.0)	1 (1.7)	1
P225H	1 (0.4)	1 (0.5)	1 (100)	1 (0.5)	0 (0.0)	1
**PI**						
Any	1 (0.4)	1 (0.5)	1 (100)	1 (0.5)	0 (0.0)	1
L90M	1 (0.4)	1 (0.5)	1 (100)	1 (0.5)	0 (0.0)	1
**Polimorphism at major resistance related positions**						
**NNRTI**						
Any	8 (3.1)	8 (3.8)	1 (12.5)	7 (3.6)	1 (1.7)	0.69
K101Q/R	6 (2.3)	6 (2.8)	1 (16.7)	5 (2.5)	1 (1.7)	1
K103Q/R	3 (1.2)	3 (1.4)	1 (33.3)	3 (1.5)	0 (0.0)	1

^a ^Mutations that have been associated with transmitted drug resistance (Shafer et al., 2007) are reported.

^b ^Number (%) of drug resistance mutations.

^c ^Number (%) of drug resistance mutations that occurs in combination with other major mutations in a sequence. The percentages were calculated in patients containing each specific mutation.

^d ^Data were analyzed by using Fisher exact test.

**Abbreviations: **PI, protease inhibitor; NRTI, nucleoside reverse transcriptase inhibitor; NNRTI, non-nucleoside reverse transcriptase inhibitor; TAMs, thymidine analogue associated mutations

Of note, 9/15 (60.0%) of these patients carried HIV-1 strains with two or more mutations associated with transmitted drug resistance. Five out of 9 patients (55.5%) were resistant to 2 drug classes (all with HIV-1 B subtype). Among these 5 patients, 4 (80.0%) were resistant to

both nucleoside reverse transcriptase inhibitors (NRTIs) and non-nucleoside reverse transcriptase inhibitors (NNRTIs), and 1 (20.0%) to NRTIs and protease inhibitors (PIs). None carried resistance to all 3 drug classes.

Major mutations associated with NRTI resistance were observed in 10 (3.9%) patients (Table 2). The revertant mutations at codon 215 were the most frequently observed (8 [3.1%]), followed by L210W (6 [2.3%]) (Table 2). Among the 8 patients with the revertant forms of T215Y/F, 5 had T215D (resulting from a single nucleotide substitution of TAC/TAT [Y] to GAC/GAT [D]), 2 had T215S (resulting from a single nucleotide substitution of either TAT/C [Y] or TTT/C [F] to TCT/TCC [S]) and 1 had a mixture of T215D/S/Y. The only TAM2 observed was D67N, found in one patient together with the TAM-1 M41L, L210W and T215Y. Similarly the 3TC-selected mutation M184V was found in only one patient (together with L210W). Thus, the majority of NRTI resistance is sustained by TAM-1 (comprising the T215Y revertant forms), suggesting that such mutational pattern confers a fitness level greater than TAM-2.

Among 9 patients (3.5%) carrying NNRTI-resistance mutations, 7 had K103N (conferring high level of resistance to both nevirapine and efavirenz) (Table 2). This mutation was found together with L100I and P225H in two and one patient, respectively; both mutations are known to occur almost exclusively with K103N in patients failing an antiretroviral regimen containing efavirenz [32].

### Association among drug resistance mutations in newly diagnosed HIV-1 infected patients

#### i) Correlated pair of mutations

A covariation analysis was performed in order to determine significant patterns of pairwise correlation between drug resistance mutations in drug-naïve patients (Table 3). The most strongly correlated pairs of major mutations in drug naïve patients are as follows: L210W+M41L (phi = 0.6) found in 3 patients, L210W+T215D (phi = 0.9) and L210W+K103N (phi = 0.4) found in 5 and 3 patients, respectively. The T215D was also strongly correlated with V60I (phi = 0.31, P = 0.02), a common polymorphism that is known to rescue the replicative capacity impaired by the TAM1 mutations in the absence of drug pressure [30].

**Table 3:** Significantly correlated pairs of mutations

Mutation 1	Frequency	Mutation 2	Frequency	Covariation Frequency	Phi^b^	*P-value*^c^
	N (%)^a^		N (%)^a^	N (%)		
**RT**						
M41L	4 (1.9)	L210W	6 (2.8)	3 (1.4)	0.6	0.02
L210W	6 (2.8)	T215D	5 (2.3)	5 (2.3)	0.9	<0.001
L210W	6 (2.8)	K103N	7 (3.3)	3 (1.4)	0.4	0.04
T215D	6 (2.8)	V60I	34 (16.0)	5 (2.3)	0.3	0.02

^*a *^The frequency was determined in 213 HIV-1 B isolates from drug-naïve patients.

^*b *^Positive correlations and negative correlations with phi >0.15 and phi <-0.07 are shown, respectively.

^*c *^All *P *values for covariation were significant at a false discovery rate of 0.05 following correction for multiple-hypothesis testing (Benjamini-Hochberg method).

#### ii) Clusters of mutations

**Figure 3:**
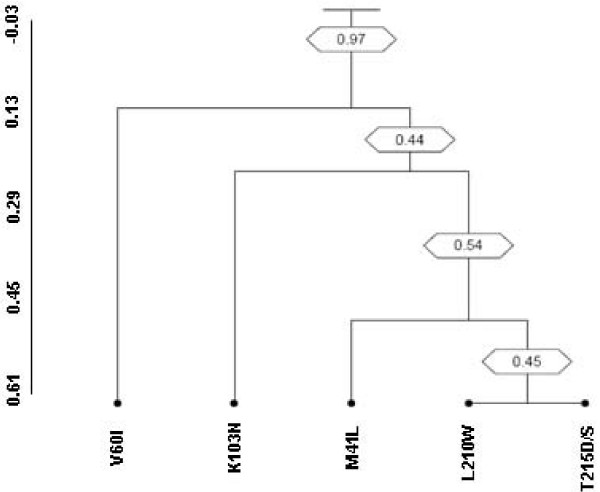
**Dendrogram obtained from average linkage hierarchical agglomerative clustering, showing significant clusters of RTI resistance mutations**. The length of branches reflects distances between mutations in the original distance matrix. Bootstrap values, indicating the significance of clusters, are reported in the boxes.

The topology of the dendrogram (Figure 3) shows the existence of one significant (bootstrap > 0.75) cluster of major mutations in newly diagnosed HIV-1 B subtype infected patients. In particular, the cluster involved four mutations associated with transmitted resistance (the TAM1 – M41L, L210W, the revertants T215D/S and the NNRTI-resistance mutation K103N) together with the polymorphism V60I (bootstrap = 0.97).

## Discussion

Our study shows that the prevalence of HIV-1 drug-resistant strains in newly diagnosed patients is 5.9%, in the range described by other European and American reports [11-16], and that the majority (60.0%) of these HIV-1 strains carries more than one major drug resistance mutation.

The prevalence of major drug resistance mutations is non-significantly different in recent infections compared to long-standing infections (3.4% *versus *6.6%); if something, a trend toward a decreased rate of resistance was found in recent infections (P = 0.09), that may reflect the increasing use of potent drugs and highly active antiretroviral regimens in recent years. At the same time, this supports the analysis of Blower that has estimated a progressive decrease in the proportion of acquired resistance from 1996 to 2005 [33]. These data suggest that the phenomenon of extensive and continuously increasing transmission of resistant strains followed by their disappearance from blood (while remaining in reservoir) is not as widespread as previously foreseen.

In addition, we found that the prevalence of mutations associated with transmitted drug resistance was higher in MSM than in heterosexual patients, in line with other European and American reports [16,34]; this factor could be related with the higher and earlier access to treatment of this class than other groups of patients.

Among major drug resistance mutations, the most common mutations observed were the NNRTI resistance mutation K103N and the NRTI T215 revertants (3.1% each one). Interestingly, the first one, K103N, was known to confer high level of resistance to nevirapine and efavirenz (without altering the viral replicative capacity), but not to the recently approved etravirine [35]. The presence of the T215 revertants suggests a previous infection with a HIV-1 strains containing T215Y/F [36,37], and has been associated with an increased risk of virologic failure in patients receiving a first line regimen with thymidine analogue [38].

We also found that about 60% of our patients harbours HIV-1 strains with more than one major drug resistance mutation. In addition, by performing a cluster analysis, we observed, in our cohort of newly diagnosed HIV-1 B subtype infected patients, the existence of a complex mutational cluster involving the revertant forms T215D/S and the TAM1 M41L, L210W (known to confer cross-resistance to all NRTIs), the K103N (known to confer cross-resistance to EFV and NVP) and the polymorphism V60I. This mutation has been shown to rescue the replicative capacity impaired by the major drug resistance mutations in the absence of drug pressure, and to contribute to the persistence over time of major drug resistance mutations in drug naïve patients [30]. Consistent with this finding, patients infected by HIV-1 strain with V60I and TAM1 had an higher viremia than those infected by HIV-1 strain with TAM1 only (5.30 *versus *3.9 log copies/ml, respectively, P = 0.09); however, the limited sample size allow us to describe only a trend, that needs to be confirmed in a larger dataset.

When we performed the clustering analysis in another cohort of 152 naïve patients from Central Italy, diagnosed between 1997–2000, we found that major mutations occurred alone in 10 (71.4%) of 14 patients with HIV-1 drug-resistant virus, more than 2 major mutations were observed only in 1 patient, and no clusters have been identified in both PR ad RT [39]. This finding is in agreement with the Spread programme [16] that showed that 71% of naïve patients with drug-resistant virus, diagnosed for HIV-1 infection between 2002 and 2003, harboured strains with only a single major mutation. Thus, two important results emerge from our study: i) newly diagnosed patients carry HIV-1 strains with more drug resistance mutations than that observed in previously diagnosed patients, ii) such mutations are organized in well defined clusters, that can seriously compromise the success of a not sufficiently potent first line regimen. These findings can have important clinical implications. In particular, our results strongly support the use of genotypic test in newly diagnosed patients. This test can help clinicians to set-up and individualize initial therapy especially in patients with extensive drug resistance. It is conceivable that in these patients the drop and long-term maintenance of viral load below 50 copies/ml can be warranted only by using a combination of potent drugs, even belonging to new class (as integrase inhibitors).

One point that is missing in most of the epidemiological studies addressing transmitted drug resistance is the global frequency of use of antiretroviral drugs (mainly NNRTI and PI) in treated patients. This may allow to compare more efficiently the different results obtained in the different countries. In addition, in the attempt to better clarify our results, we collected a cohort of 2,344 patients failing HAART regimen between 2001–2007 in Central Italy [40]. In this cohort, we observed that the percentage of patients failing an NNRTI containing regimen and the percentage of patients with NNRTI resistance mutations remained stable from 2001 to 2004, then showed a progressive decrease from 2004 to 2007 (from 40% to 26.2% and from 50% to 36.0%, respectively). This decreasing trend may be in line with the decreased percentage of drug naïve patients with NNRTI-resistance mutations (3.6% in 2004 to 0.0% in 2007). Regarding PIs, we observed that the increasing use of PIs boosted with Ritonavir (RTV) (from 18.4% in 2001 to 32.1% in 2004 and to 56.4% in 2007) is associated with a decrease in the percentage of patients with PI resistance mutations (from 58.0% in 2001 to 41% in 2004 and to 28.0% in 2007). This decreasing trend coupled with detrimental effect on viral fitness of PI resistance mutations may explain the complete absence of transmitted PI resistance mutations observed in our cohort of recently infected patients (0.0%).

Regarding the subtype distribution in our study, even if B subtype remains the prevailing one, we observed an increase of non-B subtype and of the putative recombinant forms compared to patients diagnosed in Central Italy before 2000 (16.5% vs 5.6%, P = 0.001 and 7.8% vs 0.6%, P = 0.001, respectively) (personal communication); in particular, we now report for the first time the circulation of the CRF03_AB in Italy. Of note, almost half of patients carrying non-B subtypes infected are Italian and Caucasian, confirming a diffusion and circulation of non-B subtypes within Italian population significantly increased when compared to the recent past.

## Conclusion

Our cohort of newly diagnosed HIV-1 infected patients confirms a prevalence of drug resistance mutations in line with other reports throughout Europe. As an additional information of potential clinical relevance, the increased presence of drug resistance mutations, alone or in form of complex patterns, highlights the potential risk for public health and strongly supports the extension of wide genotyping to all patients, newly diagnosed and/or patients that start an antiretroviral regimen.

## Competing interests

The authors declare that they have no competing interests.

## Authors' contributions

All of the authors participated in the establishment of the research. CA and VS participated in the design of the study, performed the statistical analysis and drafted the manuscript. CFP conceived the study, participated in its design and coordination. CG, RDA and MC carried out the molecular genetic studies. MS carried out the IgG avidity assay. SAB, FCS, MG, PE, PS and RB participated in the design of the study and helped to draft the manuscript. NO and EG contributed to the realization of the SENDIH program and participated to the coordination of this study. All authors read and approved the final manuscript.

## Pre-publication history

The pre-publication history for this paper can be accessed here:

http://www.biomedcentral.com/1471-2334/9/111/prepub

## References

[B1] Pillay D Green H Matthias R Dunn D Phillips A Sabin C Evans B UK Collaborative Group on HIV Drug Resistance Estimating HIV-1 drug resistance in antiretroviral-treated individuals in the United Kingdom J Infect Dis 2005 192 967 73 10.1086/432763 16107948

[B2] Tozzi V Zaccarelli M Bonfigli S Lorenzini P Liuzzi G Trotta MP Forbici F Gori C Bertoli A Bellagamba R Narciso P Perno CF Antinori A Collaborative Group for Clinical Use of HIV Genotype Resistance Test Drug-class-wide resistance to antiretrovirals in HIV-infected patients failing therapy: prevalence, risk factors and virological outcome Antivir Ther 2006 11 553 560 16964822

[B3] Costagliola D Descamps D Assoumou L Morand-Joubert L Marcelin AG Brodard V Delaugerre C Mackiewicz V Ruffault A Izopet J Plantier JC Tamalet C Yerly S Saidi S Brun-Vezinet F Masquelier B Agence Nationale de Recherches sur le SIDA et les Hepatites Virales (ANRS) AC11 Resistance Study Group Prevalence of HIV- drug resistance in treated patients: a French nationwide study J Acquir Immune Defic Syndr 2007 46 12 28 17514016 10.1097/QAI.0b013e318074eb73

[B4] Wensing AM Boucher CA Worldwide transmission of drug-resistant HIV AIDS Rev 2003 5 140 155 14598563

[B5] Little SJ Frost SD Wong JK Smith DM Pond SL Ignacio CC Parkin NT Petropoulos CJ Richman DD The persistence of transmitted drug resistance among subjects with primary HIV infection J Virol 2008 82 5510 5528 18353964 10.1128/JVI.02579-07 PMC2395184

[B6] Little SJ Holte S Routy JP Daar ES Markowitz M Collier AC Koup RA Mellors JW Connick E Conway B Kilby M Wang L Whitcomb JM Hellmann NS Richman DD Antiretroviral drug resistance among patients recently infected with HIV N Engl J Med 2002 347 385 394 10.1056/NEJMoa013552 12167680

[B7] Chaix ML Desquilbet L Descamps D Costagliola D Deveau C Galimand J Goujard C Signori-Schmuck A Schneider V Tamalet C Pellegrin I Wirden M Masquelier B Brun-Vezinet F Rouzioux C Meyer L French PRIMO Cohort Study Group (ANRS CO 06); French ANRS AC11 Resistance Study Group Response to HAART in French patients with resistant HIV-1 treated at primary infection: ANRS Resistance Network Antivir Ther 2007 12 1305 1310 18240870

[B8] Peuchant O Thiébaut R Capdepont S Lavignolle-Aurillac V Neau D Morlat P Dabis F Fleury H Masquelier B ANRS CO3 Aquitaine Cohort Transmission of HIV-1 minority-resistant variants and response to first-line antiretroviral therapy AIDS 2008 22 1417 1423 10.1097/QAD.0b013e3283034953 18614864

[B9] Hirsch MS Günthard HF Schapiro JM Brun-Vézinet F Clotet B Hammer SM Johnson VA Kuritzkes DR Mellors JW Pillay D Yeni PG Jacobsen DM Richman DD Antiretroviral drug resistance testing in adult HIV-1 infection: 2008 recommendations of an International AIDS Society-USA panel Clin Infect Dis 2008 47 266 285 10.1086/589297 18549313

[B10] Department of Health and Human Services Panel on Antiretroviral Guidelines for Adults and Adolescents: Guidelines for the use of antiretroviral agents in HIV-1-infected adults and adolescents 2008 1 139 http://www.aidsinfo.nih.gov/ContentFiles/AdultandAdolescentGL.pdf Accessed 12/11/2008 (page 10 and page 12, Table four, Panel's Recommendation)

[B11] Novak RM Chen L MacArthur RD Baxter JD Huppler Hullsiek K Peng G Xiang Y Henely C Schmetter B Uy J Berg-Wolf M van den Kozal M Terry Beirn Community Programs for Clinical Research on AIDS 058 Study Team Prevalence of antiretroviral drug-resistance mutations in chronically HIV-infected, treatment-naive patients: implications for routine resistance screening before initiation of antiretroviral therapy Clin Infect Dis 2005 40 468 474 10.1086/427212 15668873

[B12] Wensing AM Vijver DA van de Angarano G Asjö B Balotta C Boeri E Camacho R Chaix ML Costagliola D De Luca A Derdelinckx I Grossman Z Hamouda O Hatzakis A Hemmer R Hoepelman A Horban A Korn K Kücherer C Leitner T Loveday C MacRae E Maljkovic I de Mendoza C Meyer L Nielsen C Op de Coul EL Ormaasen V Paraskevis D Perrin L Puchhammer-Stöckl E Ruiz L Salminen M Schmit JC Schneider F Schuurman R Soriano V Stanczak G Stanojevic M Vandamme AM Van Laethem K Violin M Wilbe K Yerly S Zazzi M Boucher CA SPREAD Programme Prevalence of drug-resistant HIV-1 variants in untreated individuals in Europe: implications for clinical management J Infect Dis 2005 192 958 966 10.1086/432916 16107947

[B13] Yerly S von Wyl V Ledergerber B Böni J Schüpbach J Bürgisser P Klimkait T Rickenbach M Kaiser L Günthard HF Perrin L Swiss HIV Cohort Study Transmission of HIV-1 drug-resistance in Switzerland: a 10-year molecular epidemiology survey Antivir Ther 2007 21 2223 2229 10.1097/QAD.0b013e3282f0b685 18090050

[B14] UK Collaborative Group on HIV Drug-resistance, UK Collaborative HIV Cohort Study, UKRegister of HIV Seroconverters Evidence of a decline in transmitted HIV-1 drug-resistance in the United Kingdom AIDS 2007 21 1035 1039 17457098 10.1097/QAD.0b013e3280b07761

[B15] Payne BA Nsutebu EF Hunter ER Olarinde O Collini P Dunbar JA Basta MS Elston JW Schmid ML Thaker H Chadwick DR Low prevalence of transmitted antiretroviral drug resistance in a large UK HIV-1 cohort J Antimicrob Chemother 2008 62 464 468 10.1093/jac/dkn228 18552342

[B16] SPREAD programme Transmission of drug-resistant HIV-1 in Europe remains limited to single classes AIDS 2008 22 625 635 10.1097/QAD.0b013e3282f5e062 18317004

[B17] Orchi N Balzano R Scognamiglio P Navarra A De Carli G Elia P Grisetti S Sampaolesi A Giuliani M De Filippis A Puro V Ippolito G Girardi E SENDIH group Ageing with HIV: newly diagnosed older adults in Italy AIDS Care 2008 20 419 425 10.1080/09540120701867073 18449818

[B18] Suligoi B Massi M Galli C Sciandra M Di Sora F Pezzotti P Recchia O Montella F Sinicco A Rezza G Identifying recent HIV infections using the avidity index and an automated enzyme immunoassay J Acquir Immune Defic Syndr 2003 32 424 428 10.1097/00126334-200304010-00012 12640201

[B19] Selleri M Orchi N Zaniratti MS Bellagamba R Corpolongo A Angeletti C Ippolito G Capobianchi MR Girardi E Effective highly active antiretroviral therapy in patients with primary HIV-1 infection prevents the evolution of the avidity of HIV-1-specific antibodies J Acquir Immune Defic Syndr 2007 46 145 150 10.1097/QAI.0b013e318120039b 17589369

[B20] Suligoi B Buttò S Galli C Bernasconi D Salata RA Tavoschi L Chiappi M Mugyenyi P Pimpinelli F Kityo C Regine V Rezza G Detection of recent HIV infections in African individuals infected by HIV-1 non-B subtypes using HIV antibody avidity J Clin Virol 2008 41 288 92 10.1016/j.jcv.2007.11.020 18248848

[B21] Ceccherini-Silberstein F Erba F Gago F Bertoli A Forbici F Bellocchi MC Gori C D'Arrigo R Marcon L Balotta C Antinori A Monforte AD Perno CF Identification of the minimal conserved structure of HIV-1 protease in the presence or absence of drug pressure AIDS 2004 18 11 19 10.1097/01.aids.0000131394.76221.02 15280771

[B22] Thompson JD Higgins DG Gibson TJ Clustal W: Improving sensitivity of progressive multiple sequence alignment through sequence weighting, position-specific gap penalties and weight matrix choice Nucl Acids Res 1994 22 4673 4680 7984417 10.1093/nar/22.22.4673 PMC308517

[B23] Hall TA Bioedit: a user-friendly biological sequence alignment editor and analysis program for Windows 95/98 NT Nucl Acids Symp Ser 1999 41 95 98

[B24] de Oliveira T Deforche K Cassol S Salminen M Paraskevis D Seebregts C Snoeck J van Rensburg EJ Wensing AM Vijver DA van de Boucher CA Camacho R Vandamme AM An automated genotyping system for analysis of HIV-1 and other microbial sequences Bioinformatics 2005 21 19 3797 800 10.1093/bioinformatics/bti607 16076886

[B25] Swafford KL PAUP 4,0: Phylogenetic analysis with parsimony (and other methods), version 4,0b2a 1999 Sinauer Associates Inc., Sunderland, MA

[B26] Posada D Crandall KA MODEL TEST: Testing the model of DNA substitution Bioinformatics 1998 14 817 818 10.1093/bioinformatics/14.9.817 9918953

[B27] Ray S Simplot v3.2 beta 2002 Baltimore, MD distributed by author

[B28] Shafer RW Rhee SY Pillay D Miller V Sandstrom P Schapiro JM Kuritzkes DR Bennett D HIV-1 protease and reverse transcriptase mutations for drug-resistance surveillance AIDS 2007 21 215 223 17197813 10.1097/QAD.0b013e328011e691 PMC2573394

[B29] Shafer RW Rhee SY Bennett DE Consensus drug resistance mutations for epidemiological surveillance: basic principles and potential controversies Antivir Ther 2008 13 59 68 18575192 PMC4388302

[B30] Lindström A Ohlis A Huigen M Nijhuis M Berglund T Bratt G Sandström E Albert J HIV-1 transmission cluster with M41L 'singleton' mutation and decreased transmission of resistance in newly diagnosed Swedish homosexual men Antivir Ther 2006 11 1031 1039 17302373

[B31] Svicher V Sing T Santoro MM Forbici F Rodríguez-Barrios F Bertoli A Beerenwinkel N Bellocchi MC Gago F d'Arminio Monforte A Antinori A Lengauer T Ceccherini-Silberstein F Perno CF Involvement of novel human immunodeficiency virus type 1 reverse transcriptase mutations in the regulation of resistance to nucleoside inhibitors J Vir 2006 80 7186 7198 10.1128/JVI.02084-05 PMC1489024 16809324

[B32] Bacheler LT Anton ED Kudish P Baker D Bunville J Krakowski K Bolling L Aujay M Wang XV Ellis D Becker MF Lasut AL George HJ Spalding DR Hollis G Abremski K Human immunodeficiency virus type 1 mutations selected in patients failing efavirenz combination therapy Antimicr Agents Chemother 2000 44 2475 8244 10.1128/AAC.44.9.2475-2484.2000 PMC90088 10952598

[B33] Blower SM Aschenbach AN Gershengorn HB Kahn JO Predicting the unpredictable: transmission of drug-resistant HIV Nat Med 2001 7 1016 1020 10.1038/nm0901-1016 11533704

[B34] Weinstock HS Zaidi I Heneine W Bennett D Garcia-Lerma JG Douglas JM Jr LaLota M Dickinson G Schwarcz S Torian L Wendell D Paul S Goza GA Ruiz J Boyett B Kaplan JE The epidemiology of antiretroviral drug-resistance among drug naive HIV-1-infected persons in 10 US cities J Infect Dis 2004 189 2174 2180 10.1086/420789 15181563

[B35] Vingerhoets J Buelens A Peeters M Picchio G Tambuyzer L Cao Van K De Smedt G Woodfall B de Bethune MP Impact of baseline NNRTI mutations on the virological response to TMC125 in the phase III clinical trials DUET-1 and DUET-2 Antivir Ther 2007 12 suppl S34

[B36] de Ronde A van Dooren M Hoek L van Der Bouwhuis D de Rooij E van Gemen B de Boer R Goudsmit J Establishment of new transmissible and drug-sensitive human immunodeficiency virus type 1 wild types due to transmission of nucleoside analogue-resistant virus J Vir 2001 75 595 602 10.1128/JVI.75.2.595-602.2001 PMC113955 11134272

[B37] Garcia-Lerma JG Nidtha S Blumoff K Weinstock H Heneine W Increased ability for selection of zidovudine resistance in a distinct class of wild-type HIV-1 from drug-naive persons Proc Nat Acad Sc USA 2001 98 13907 13912 10.1073/pnas.241300698 PMC61140 11698656

[B38] Violin M Cozzi-Lepri A Velleca R Vincenti A D'Elia S Chiodo F Ghinelli F Bertoli A d'Arminio Monforte A Perno CF Moroni M Balotta C Risk of failure in patients with 215 HIV-1 revertants starting their first thymidine analog-containing highly active antiretroviral therapy AIDS 2004 18 227 235 10.1097/00002030-200401230-00012 15075540

[B39] Alteri C Svicher V Gori C D'Arrigo R Santoro MM Bellocchi MC Ceccherini-Silberstein F Orchi N Girardi E Perno CF Complex patterns of drug-resistance mutations in newly-diagnosed HIV-infected patients: implications with the transmission of resistant strains Presented at 6th European HIV Drug-resistance Workshop 2008 Abstract 13

[B40] Santoro MM Ceccherini-Silberstein F Narciso P Mussini C Zaccarelli M Borghi V Boumis E Tozzi V Visco-Comandini V Gori C Forbici F d'Arrigo R Bellocchi MC Bertoli A Antinori A Perno CF Declining of HIV-1 drug resistance in treatment-failing patients: a potential association with more effective antiretroviral regimens Presented at 6th European HIV Drug Resistance Workshop 2008 Abstract 2

